# Chitosan-Bead-Encapsulated Polystyrene Sulfonate for Adsorption of Methylene Blue and Regeneration Studies: Batch and Continuous Approaches

**DOI:** 10.3390/polym15051269

**Published:** 2023-03-02

**Authors:** Kam Sheng Lau, Nur Alia Sahira Azmi, Siew Xian Chin, Sarani Zakaria, Chin Hua Chia

**Affiliations:** 1 Materials Science Program, Department of Applied Physics, Faculty of Science and Technology, Universiti Kebangsaan Malaysia, Bangi 43600, Selangor, Malaysia; 2 ASASIpintar Program, Pusat GENIUS@Pintar Negara, Universiti Kebangsaan Malaysia, Bangi 43600, Selangor, Malaysia

**Keywords:** adsorption isotherm, dye removal, polyelectrolyte, regeneration

## Abstract

Textile industrialization causes water pollution due to the discharge of industrial effluents into the environment. To reduce the impact of industrial effluent, it must be treated in wastewater treatment plants before discharge into rivers. Among all wastewater treatment approaches, the adsorption process is one method to remove pollutants from wastewater, but it has some limitations in term of reusability and ionic selective adsorption properties. In this study, we prepared cationic poly (styrene sulfonate) (PSS)-incorporated anionic chitosan beads synthesized using the oil–water emulsion coagulation method. The produced beads were characterized using FESEM and FTIR analysis. In batch adsorption studies, the PSS-incorporated chitosan beads exhibited monolayer adsorption processes, that is, exothermic processes that occur spontaneously at low temperatures, which were analyzed based on the adsorption isotherms, adsorption kinetics, and thermodynamics model fittings. The presence of PSS enables cationic methylene blue dye to adsorb to the anionic chitosan structure via electrostatic interaction between the sulfonic group and the dye molecule. The maximum adsorption capacity of PSS-incorporated chitosan beads achieved 42.21 mg/g, as calculated from the Langmuir adsorption isotherm. Finally, the PSS-incorporated chitosan beads demonstrated good regeneration with different types of reagents, especially using sodium hydroxide as a regeneration reagent. With the use of sodium hydroxide regeneration of this adsorbent material, a continuous adsorption setup also demonstrated that PSS-incorporated chitosan beads can be reused for methylene blue adsorption for up to three cycle processes.

## 1. Introduction

Active industrialization activities, such as production, pharmaceutical production, textiles, chemical synthesis, tanning, battery production, and agriculture, have generated heavy metals and organic pollutants that are harmful and toxic to the ecosystem [1]. One of the main contaminants in industrial effluents that seriously pollutes water is dye [2]. An estimated 1 million tons of dyes are produced annually to satisfy the needs of various sectors, including textile, paper, leather, and others. Over 54% of all dye wastewater is produced by the textile sector [3]. Dye effluents have received a lot of attention recently because of the harm they do to the ecosystem as water pollution in terms of visual character, carcinogenicity, and accumulation in organisms. The presence of dyes in water prevents sunlight from reaching aquatic plants, toxins from degraded goods make water poisonous, and the CODs of sources of water increase [4]. Therefore, researchers have created several technologies for the purification and removal of contaminants from wastewater prior to its release into the sewage system to solve the problem of water pollution. Conventionally, wastewater treatment uses techniques such as coagulation and flocculation; however, the adsorption process is an alternative method to remove pollutants from wastewater [5,6].

Researchers have used several materials to remove toxic organic compounds and heavy metal including metal oxides, metal–organic frameworks, cellulose nanofibrils, chitosan composites, etc. [7,8,9,10,11] To further improve the adsorption properties of adsorbent materials, studies have shown that the usage of polyelectrolyte, added into wastewater, proceeds to interact with ionic compounds such as heavy metals and cationic or anionic compounds [12,13]. Polyelectrolytes are an intriguing class of macromolecules known as a form of polymer with dissociated ionic groups [13,14]. The natural interaction of polyelectrolytes involves reversible interaction between anionics and cationic groups and depends on the properties of the reacting polyelectrolyte. [14] There are many types of polyelectrolytes that can be classified into natural, chemically modified, or synthetic type and categorized as different types based on their origin, charge (cationic type, e.g., polyethyleneimine, polyallylamine hydrochloride and anionic type, e.g., polyacrylic acid, poly sodium styrene sulfonate, polyvinyl sulfate, polydopamine-g-l-cysteine), charge density, composition, position of ion site, and shape [14,15].

The benefit of charge polyelectrolytes is their ability to interact strongly with oppositely charged surfaces and macromolecules. However, due to the solubility of these materials in water, making them difficult to recover, polyelectrolyte recycling and reuse have still not been accomplished. Therefore, these polyelectrolytes can be encapsulated to form porous composites with natural biopolymers such as cellulose, chitosan, gelatin, carrageenan, etc. [11,16,17,18,19]. Chitin is an insoluble linear polysaccharide, made of β-1,4-N-acetylglucosamine, which has high crystalline structure and intermolecular hydrogen bonding [20]. Typically, chitin sources can be obtained from discarded crustacean shells from the seafood processing sector, such as crab or shrimp shells; additionally, it can also be found in many microbes such as algae, fungi, and protists [21]. Furthermore, chitin can be converted into chitosan through a deacetylation reaction by heating it with alkaline solution. Chitosan comes in a variety of forms such as nanofibers, sponges, gel, beads, scaffolds, and films [11,22]. Chitosan can also be applied in various applications such as in the biomedical and food industries due to its excellent biological activities such as anti-oxidant, anti-inflammatory, hemocompatible, anti-fungal, and anti-microbiological properties [23].

Adsorbent material regeneration is essential for reducing process costs, recovering resources, avoiding secondary contamination, reducing the need to manufacture new adsorbent, and recovering extracted contaminants from aqueous solution. The adsorbed adsorbate molecules must be desorbed to regenerate and reuse the adsorbent for another cycle [24]. Previous research on polyelectrolyte-based adsorbent materials was conducted by Zheng et al. (2020) [25]. They studied the adsorption behaviors of novel magnetic adsorbent FS@CS-PAA prepared by coating anionic polyacrylamide-modified chitosan (CS-PAA) on the surface of silica-combined Fe3O4 (FS) towards cationic dyes and metal ions. These experiments were performed in single systems, binary systems, and model wastewater. After treatment through synthetic wastewater and model wastewater, FS@CS-PAA was regenerated using HCl solution.

Zhao et al. (2020) studied polyelectrolyte complex hydrogels (PECHs) prepared with polyampholyte polysaccharides. The PECHs of chitosan (CTS) and carboxymethylcellulose were prepared as semi-dissolution acidification sol–gel transition product [26]. The hydrogel was formed by the strong electrostatic interactions of the cationic -NH3^+^ groups of CTS and the anionic –COO^−^ groups of CMC. The PECHs prepared had good adsorption capacity for both cationic and anionic dyes, with maximum adsorption capacity of 212.83 mg/g for sunset yellow FCF and 167.35 mg/g for methylene blue. A novel adsorbent acrolein crosslinked polyethylenimine–chitosan hydrogel (A-PEI/CS) was developed by Wan et al. (2022) with remarkable recycling stability and ion-enhanced effect for removing anionic acid blue 93 dye from an aquatic environment [27]. It had high adsorption capacity and a continuous recycling ability of more than 15 times. The elimination rate of A-PEI/CS for acid blue 93 could still be as high as 96% with a barely perceptible decreasing trend after 15 desorption–regeneration cycles. Yin et al. (2023) investigated methacrylated alginate (AlgMA) and sodium p-styrenesulfonate (NaSS), focusing on covalently crosslinked microporous cryogels capable of effectively removing cationic dyes and fabricated using the freezing radical copolymerization method to form cryogel [28]. They found that the the cryogel samples demonstrated higher adsorption of methylene blue under alkaline conditions. The AlgMA/PNaSS cryogels also exhibited selective adsorption capacity and reusability following regeneration, and their corrected adsorption capacity remained unaffected over the five adsorption–desorption cycles.

Furthermore, adsorption studies have been performed by researchers in batch, up-flow, or down-flow continuous fixed bed, continuous fluidized bed, continuous moving bed and oscillated (pulsed) bed studies, of which batch and up-flow continuous fixed bed adsorption investigations were most feasible due to easy and cheap techniques that could remove most of the contaminations used in a pilot wastewater treatment plant that used a continuous wastewater treatment approach [29,30]. Jain et al. (2020) investigated batch adsorption using waste tea residue (WTR) to adsorb Acid Blue 25 dye with a maximum dye absorption of 127.14 mg g^−1^ at an optimum pH of 1 and a loading of 3.5 g L^−1^ [2]. Meanwhile, continuous investigations performed in packed columns with column operating parameters such as packing height (3–6 cm), concentration (50–200 mg L^−1^) and influent flow rate (5–9 mL min^−1^) on the efficiency of dye remediation revealed a maximum absorption of 50.82 mg g^−1^. The findings of batch and continuous investigations show that WTR can be used successfully to remove a specific anionic dye from the aqueous phase. Patel (2020) also reported batch and continuous fixed bed adsorption of heavy metals using activated charcoal, which achieved adsorption capacities of 205.6, 185.8, 154.5, 133.3, 120.6, and 110.9 mg/g for Pb, Cu, Cd, Zn, Ni, and Cr, respectively. The activated carbon could also be regenerated using hydrochloric acid and reused for seven consecutive cycles. Moreover, Marsiezade and Javanbakht (2020) prepared carboxymethyl cellulose-based ZSM-5/zeolitic imidazolate framework (CMC/ZSM-5/ZIF-8) hollow beads for adsorption of methylene blue in batch and continuous fixed bed systems that could be regenerated using distilled water and ethanol [31]. In a batch equilibrium adsorption study, they reported that the CMC/ZIF-8 beads demonstrated the highest adsorption capacity of 13.06 mg g^−1^ compared with other samples, whereas in continuous adsorption studies it also demonstrated the highest capacity (11.87 mg g^−1^) compared with other samples.

In this study, PSS was incorporated into chitosan beads in order to adsorb cationic methylene blue dyes. The chitosan beads were characterized with a field emission scanning electron microscope (FESEM) and Fourier transform infrared (FTIR) spectroscopy. The adsorption properties of the PSS incorporated chitosan beads were studied in batch and continuous studies. Various adsorption kinetics and an adsorption isotherm model were implemented on the results to explain the adsorption behavior of the adsorbent material. Adsorbent regeneration studies were conducted using various reagents to select the best desorption agent to recycle the chitosan beads.

## 2. Materials and Methods

### 2.1. Materials

Ethyl acetate (>99.5%, EMSURE^®^ ACS), glacial acetic acid (100%, EMSURE^®^ ACS), hydrochloric acid (HCl, 37%, EMSURE^®^ ACS), oxalic acid dihydrate (99.5%, EMSURE^®^ ACS), pyridine (>99.5%, EMSURE^®^ ACS), sodium hydroxide (>99%, EMSURE^®^ ACS) and urea (99%, ACS) were purchased from Merck Millipore (Burlington, MA, USA). Chitosan (medium molecular weight, M_w_ = 190,000–310,000 g/mol based on viscosity), methylene blue (ACS reagent), and poly(sodium 4-styrenesulfonate) solution (Mw ~70,000, 30 wt% in H_2_O) were purchased from Sigma Aldrich (Saint Louis, MO, USA).

### 2.2. Preparation of Chitosan and Chitosan-PSS (Chi-PSS) Beads

**Scheme 1:**
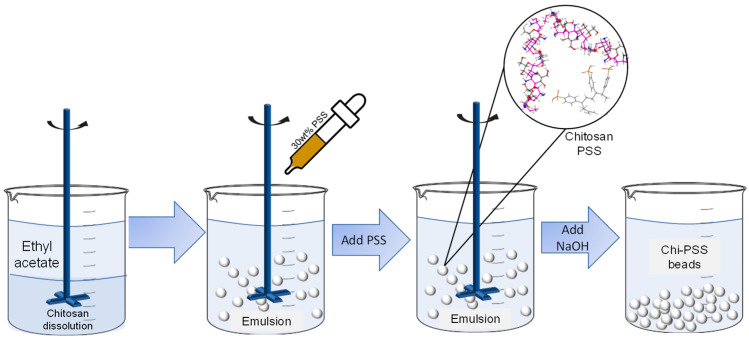
Illustration of the preparation of Chi-PSS beads via emulsion coagulation method.

Chitosan (2 g) was dissolved in 100 mL of 2.5 wt% acetic acid (2.5 g) solution in deionized water by stirring at 80 °C for one hour until the chitosan powder fully dissolved [32]. Then, 50 mL of chitosan dissolution was added into 50 mL of ethyl acetate and stirred with an overhead stirrer for 30 min until an emulsion was formed. Then, PSS solution was added into the emulsion and mixed for another 30 min. To form beads, 1 M of NaOH solution was added into the chitosan-PSS emulsion and the solution was stirred for 1 h to allow coagulation as shown in Scheme 1. After one hour of coagulation, the chitosan beads were collected and then washed repeatedly with distilled water to remove the ethyl acetate. For the chitosan beads, the steps were repeated without the addition of PSS solution.

### 2.3. Characterisation of Chitosan and Chi-PSS Beads

Chitosan bead surface morphology and cross-section structure were examined using a field emission scanning electron microscope (FESEM, FEI Quanta 400F, Hillsboro, OR, USA). Fourier transform infrared (FTIR) spectra were obtained using an attenuated total reflection (ATR) mode infrared spectrometer (Bruker Alpha, Ettlingen, Germany).

### 2.4. Adsorption Isotherms, Kinetics, and Thermodynamics

Methylene blue solution was used as a model cationic dye without further purification. A stock solution of methylene blue (500 mg/L) was prepared and diluted to the following concentrations: 25, 50, 100, 200, and 400 mg/L. The absorbance of methylene blue was measured at 660 nm using a UV-vis spectrophotometer (Jenway 7315, Stone, UK) to construct a standard calibration curve according to Beer–Lambert’s law. For each run, 0.1 g of beads was added into 50 mL of the methylene blue solution with stirring using a magnetic stirrer. For batch adsorption studies, the mass of adsorbent (0.05, 0.1, 0.25, 0.5 g) and effect of temperature (25, 45, 65 °C) were studied. In addition, 0.1 g of adsorbed beads was regenerated using 100 mL of 0.1 M of chemical reagents (pyridine, urea, oxalic acid, HCl, NaOH) for 1 h and the beads were rinsed with distilled water to remove the reagent. For continuous adsorption studies, 0.1 g of beads was packed in a fixed bed column and 15 mg/L methylene blue dye solution was pumped into the column with a flow rate of 1 mL/min; the results were plotted as a breakthrough curve.

## 3. Results

### 3.1. Characterization

**Figure 1:**
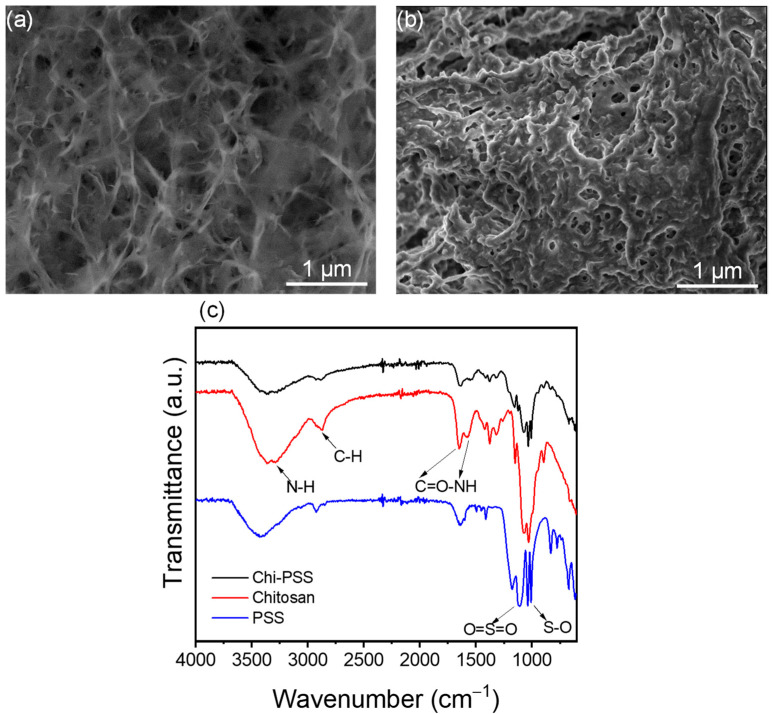
FESEM images of the cross-section view of (**a**) chitosan beads and (**b**) Chi-PSS beads. (**c**) FTIR spectra of the chitosan, Chi-PSS, and PSS polymer.

The morphological images of the chitosan beads with and without PSS are shown in Figure 1a,b. The chitosan beads show a porous networking in their internal structure. With the incorporation of PSS, the pore surface shows a rougher structure due to the coagulation of PSS polymer chains on the chitosan backbone structure due to the electrostatic interaction between them. Additionally, the average pore sizes estimated from FESEM images for chitosan and Chi-PSS were 37 ± 30 and 104 ± 31 nm, respectively. Furthermore, the FTIR spectra of the chitosan, Chi-PSS, and PSS polymer are displayed in Figure 1c. Several peaks correspond to functional groups in the glucosamine structure of the chitosan backbone chain, namely those at 3285 cm^−1^ (N–H stretching), 2929 cm^−1^ (C–H stretching), 1650 and 1572 cm^−1^ (C=O and N–H stretching of amide group), 1377 cm^−1^ (primary alcohol), and 1028 cm^−1^ (C–O–C stretching) [33]. Meanwhile, the peaks corresponding to the sulfonate group presence in the PSS are 1412 and 1110 (sulfonic acid), 1038 (sulfoxide) and 775 cm^−1^ (C–H in the styrene) [34].

### 3.2. Adsorption Kinetics

To study the adsorption mechanism of interaction between the Chi-PSS beads and the methylene blue dye molecules as well as potential rate-determining steps, some conventional kinetic models were evaluated by using three commonly used kinetic models: the pseudo-first order, pseudo-second order and intra-particle diffusion models. The adsorption experiments were conducted with different amounts of adsorbent (0.05, 0.1, 0.25 and 0.5 g) at 25 °C.

In general, the pseudo-first order model follows Lagergren’s kinetics and describes the adsorption process as a physisorption. Meanwhile, the pseudo-second order model describes adsorption as a chemisorption process. Lastly, the intra-particle diffusion model follows the Weber–Morris model and describes pore diffusion in adsorption processes.

The equation for the pseudo-first order kinetic model is described below [35]:

ln (q_e_ − q_t_) = ln (q_e_) − k_1_t (1)

q_t_ (mg/g) and q_e_ (mg/g) are the amount of methylene blue adsorbed at time t and equilibrium, and k_1_ is the first order-kinetic constant. The value of k_1_ was obtained from the slope, plotting ln (q_e_ − q_t_) against t.

The equation for the pseudo-second order kinetic model is described as below [36]:

1/q_t_ = 1/(k_2_q_e_^2^t) + 1/q_e_ (2)

where k_2_ is the pseudo-second order kinetic constant. The value of k_2_ was calculated from the slope (1/k_2_q^2^_e_) and intercept (1/q_e_) when 1/q_t_ was plotted against 1/t.

The equation for intraparticle diffusion kinetic model is described below:

q_t_ = K_p1_t^1/2^ + C (3)

where K_p1_ (mg g^−1^ min^−1/2^) is the rate coefficient of intraparticle diffusion and C describes the constant thickness of the boundary layer. The value of K_p1_ was calculated from the slope from when qt was plotted against t^1/2^.

**Figure 2:**
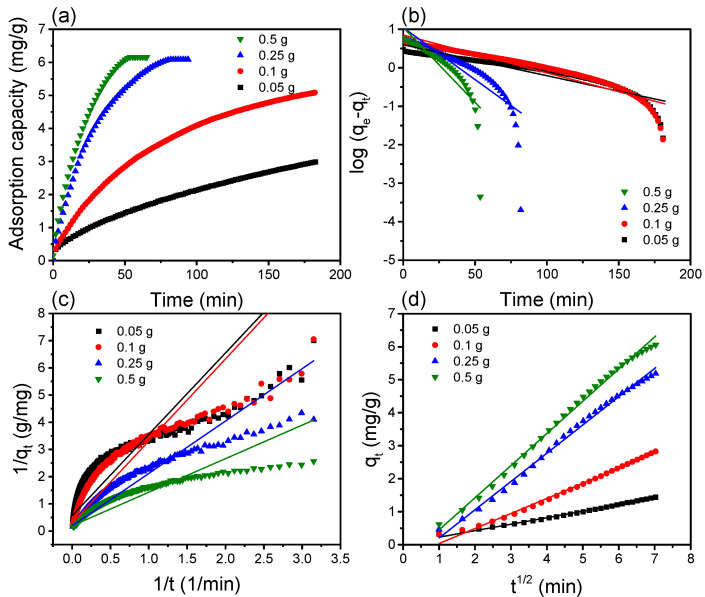
(**a**) Adsorption kinetics of the Chi-PSS beads and plot of (**b**) pseudo-first order adsorption kinetics, (**c**) pseudo-second order adsorption kinetics, (**d**) intraparticle diffusion with adsorption of 15 mg/L methylene blue at various loading of adsorbent.

**Scheme 2:**
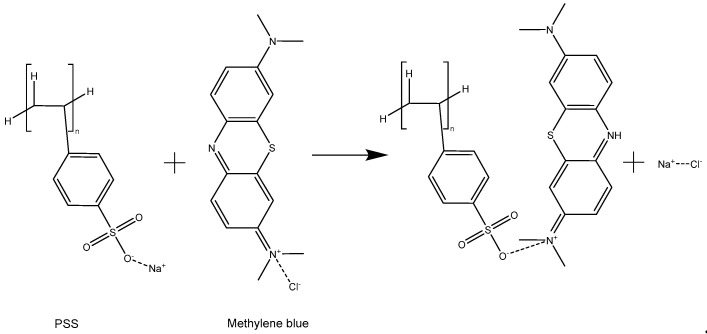
Illustration of adsorption process via electrostatic interaction (dashed line) between the cationic dye molecule and anionic sulfonate group in the PSS.

The adsorption kinetics of the Chi-PSS beads and kinetic model fitted plot are shown in Figure 2. Table 1 shows the kinetics rate constants and regression coefficients for each model. The overall regression coefficient is higher in the pseudo-first order kinetic model compared to the pseudo-second order kinetic model, indicating that the adsorption process of Chi-PSS is physisorption via electrostatic interaction between the cationic dye molecule and anionic sulfonate group in the PSS (Scheme 2). Additionally, the intraparticle diffusion shows an increasing ratio of adsorbent. However, the regression coefficient for the pseudo-first order model is slightly lower than the two other models, indicating that the adsorption process could be validly described by the pseudo-second order kinetic model. The intraparticle model shows only one fitted plot, indicating that initial adsorption behavior remains that same throughout the adsorption process until reaches equilibrium [37]. Additionally, the diffusion rate increases when the mass of adsorbent increases due to the adsorption process involving pore diffusion of dye molecules in the Chi-PSS beads [38]. As the ratio of pore in Chi-PSS to methylene blue molecules increases, the available active sites of pores also increase. Furthermore, the adsorption capacity calculated based on the pseudo-second order kinetic model was also matched the trend of the adsorption capacity estimated from the graph.

### 3.3. Adsorption Isotherm and Thermodynamic Analysis

The equilibrium isotherm is used for designing and characterizing the adsorption-based separation process. It describes the interaction between adsorbent and adsorbate under certain conditions. With recorded equilibrium data, this experiment used fundamental isotherm models such as Langmuir, Freundlich, and the Temkin model [39]. In general, the Langmuir model considers the adsorption and desorption rates to be the same in the equilibrium, and the adsorption equilibrium constant is obtained. The Freundlich model approach is an empirical adsorption model stating that surface coverage has no effect on the adsorption energy on a homogeneous surface. The Temkin model assumes that with increasing surface coverage of adsorbent, the adsorption heat of all molecules falls linearly.

The equation for the Langmuir adsorption isotherm model is shown below:

q_e_ = q_m_K_L_C_e_/(1 + K_L_C_e_) (4)

where q_e_ is the amount of methylene blue adsorbed on the Chi-PSS beads at equilibrium (mg/g) and indicates the adsorption capacity at equilibrium and C_e_ is the equilibrium concentration of the dye, while q_m_ is the maximum adsorption capacity of methylene blue (mg/g) and K_L_ is Langmuir’s adsorption equilibrium constant (L/mg).

The equation for the Freundlich adsorption isotherm model is shown below:

q_e_ = K_F_C_e_^1/n^ (5)

where q_e_ is the amount of methylene blue adsorbed on the Chi-PSS beads at equilibrium (mg/g) and indicates the adsorption capacity at equilibrium, C_e_ is the equilibrium concentration of the dye, K_F_ is the distribution coefficient of methylene blue on the adsorbent surface, and n is the correction factor.

The equation for the Temkin adsorption isotherm model is shown below:

q_e_ = (RT Ln(K_T_C_e_))/b_T_ (6)

where q_e_ is the amount of methylene blue adsorbed on the Chi-PSS beads at equilibrium (mg/g) and indicates the adsorption capacity at equilibrium, C_e_ is the equilibrium concentration of the dye, q_m_ is the maximum adsorption capacity of methylene blue (mg/g), and K_L_ is Langmuir’s adsorption equilibrium constant (L/mg).

**Figure 3:**
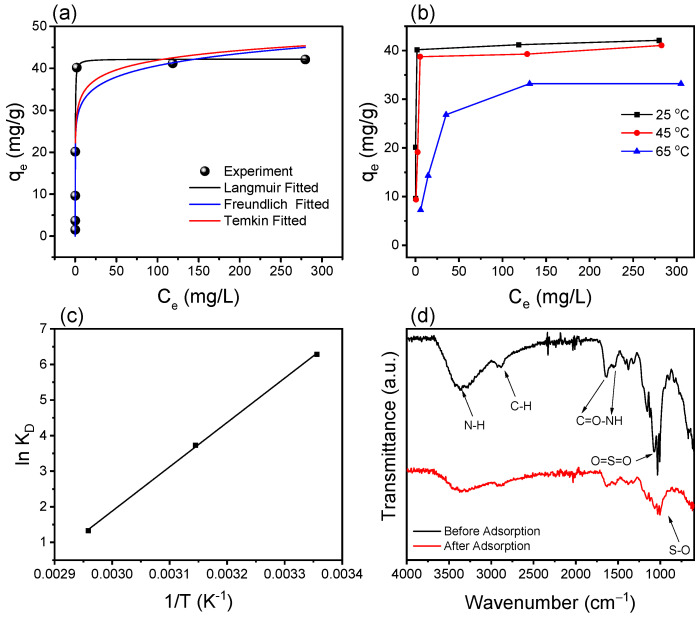
(**a**) q_e_ vs. C_e_ plot for Chi-PSS bead fitted with Langmuir, Freundlich, and Temkin isotherm models. (**b**) q_e_ vs. C_e_ plot and (**c**) Van’t Hoff’s plot for methylene blue adsorption of Chi-PSS beads at various temperatures. (**d**) FTIR spectra of the Chi-PSS beads after methylene blue adsorption.

For the best fitting of data, the non-linear fitting method was applied, and the correlation coefficients (r^2^) were calculated to compare the degrees of accuracy of the models. Figure 3a shows the plot of (q_e_) the amount of the methylene blue at equilibrium against (c_e_) the equilibrium concentration of methylene blue on the Chi-PSS beads (mg/L) over the three models used in this experiment, and the fitted results are tabulated in Table 2. Based on adsorption isotherm model fitting, the highest value of (r^2^) was recorded for the fit of the Langmuir model compared to the Freundlich and Temkin models, with values of 0.97, 0.85, and 0.88, respectively; this indicates that adsorption was favorable and perfectly suits the Langmuir isotherm model compared to the others. The maximum adsorption capacity (Q_0_) calculated from the Langmuir adsorption isotherm model for Chi-PSS beads is 42.21 mg/g. The Langmuir adsorption constant (K_L_) for this experiment is 6.36. A variation of the suitable area and the adsorbent’s porosity can be correlated with the K_L_ constant, implying that higher adsorption capacity can result from larger surface area and pore volume of Chi-PSS beads.

According to Langmuir isotherm model fitting theory, it is assumed that adsorption of methylene blue on Chi-PSS beads occurs in monolayer adsorption, where it is adsorbed as a one-molecule layer at equivalent localized sites [40]. Additionally, the PSS coated on chitosan forms a solid homogenous surface and there is no steric hindrance and lateral interaction between adjacent sites, that is, between the adsorbed molecules [40]. This means that once methylene blue molecule occupies an active site, no further adsorption by other methylene blue molecules occurs in the process.

Thermodynamic analysis can be used to determine the feasibility and nature of adsorption processes [41]. Thermodynamics parameters such as change in Gibbs free energy (ΔG°), change in enthalpy (ΔH°), and change in entropy (ΔS°) were evaluated to predict the feasibility of the adsorption. The values of ΔH and ΔS were measured using the Van’t Hoff equation. In this study, the effect of different temperatures with various concentrations of methylene blue on Chi-PSS adsorption was studied.

The equation for thermodynamic parameters is shown below [42,43]:

K_e_° = K_L_ [absorbate]^0^/γ (7)

ΔG =−RT ln K_e_° (8)

Ln K_e_° =−ΔH°/RT + ΔS°/R (9)

where R is the gas constant (8.3145 J K^−1^ mol^−1^), T is the temperature in (Kelvin), K is the equilibrium constant, K_e_° (dimensionless) is the adsorption equilibrium constant, K_L_ (L·mol^−1^) is the Langmuir equilibrium constant, [absorbate]^0^ (mol·L^−1^) is the standard concentration of adsorbate, and γ (dimensionless) is the coefficient of activity.

Figure 3b shows the effect of temperature on the adsorption isotherm of the Chi-PSS beads. Along the increase in temperature from 25 to 65 °C, the q_e_ value can be seen to achieve the optimum rate of adsorption only until 45 °C, slowly dropping as the temperature reaches 65 °C. Based on this study, increasing the concentration of methylene blue using the adsorption data obtained under the different temperatures and different concentrations of methylene blue, a Van’t Hoff plot of ln K against 1/T was plotted as shown in Figure 3c and the thermodynamic parameters calculated from the fitted plot is tabulated in Table 3. It can be seen that the graph shows positive slope, indicating that the adsorption process of methylene blue in Chi-PSS is exothermic in nature. The value of (ΔH°) shows a negative result, confirming the exothermic nature of this study. However, there is a slow decrease in negative value with increasing concentration. The negative value of ΔS° shows that the interaction of methylene blue dye molecules on the surface of Chi-PSS during the adsorption process is non-random. The positive value of ΔG° with increasing temperature and negative value of ΔS° indicates that the adsorption of methylene blue is non-favored with increasing temperature. In addition, the FTIR analysis of the Chi-PSS beads after the adsorption of methylene blue is shown in Figure 3d. As the Chi-PSS beads adsorb the methylene blue molecules, the chemical bonding for O=S=O and S-O shows a decrease in transmittance, which could be due to the interaction between the sulfonate group and cationic dye molecules that reduce the polarity of the structure.

### 3.4. Adsorbent Regeneration Studies and Continuous Adsorption Process

**Figure 4:**
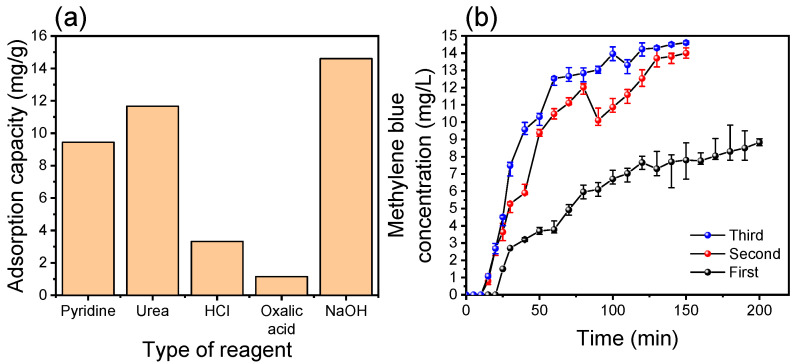
(**a**) Adsorption capacity of the Chi-PSS beads after regeneration with various chemical reagents. (**b**) Breakthrough curves for Chi-PSS beads after regeneration using NaOH for 3 consecutive cycles.

The evaluation of Chi-PSS bead regeneration and recyclability is illustrated in Figure 4a. The results show that the second adsorption cycle for Chi-PSS beads treated with 0.1 M NaOH has higher adsorption performance compared to pyridine, urea, HCl and oxalic acid. This could be due to the influence of pH on the NaOH solution weakening the electrostatic interactions between dye molecules and the adsorption sites on the adsorbents, causing the desorption of cationic dye molecules from the PSS surface as a result of the higher affinity of the cationic parts of the desorption agents (Na^+^) when electrostatically interacting with the anionic sulfonic groups in the PSS structure. Based on the electrostatic charge interactions between PSS and methylene blue molecules proposed in Scheme 2, this could be due to the sulfonate group in PSS having higher selectivity with Na^+^ ions compared to other counter ions. The decrease in number of charged functional groups on the adsorbent may result in electrostatic repulsion and desorption of cationic dye molecules.

After understanding the adsorption behavior of the Chi-PSS beads in the batch adsorption setup, the Chi-PSS was then packed into a fixed bed column to study its adsorption performance under a continuous flow of methylene blue solution. For the continuous adsorption study (Figure 4b), the Chi-PSS beads removed all the methylene blue dye during the first 20 min in the first cycle. The Chi-PSS beads were regenerated with NaOH after each adsorption cycle and the adsorption capacities for the first, second, and third cycles were 94.17, 59.33, and 44.45 mg/g, respectively. The Chi-PSS beads were able to perform adsorption up to the third cycle, signifying the reusability of Chi-PSS for methylene blue adsorption.

## 4. Conclusions

The cationic chitosan beads incorporated with anionic PSS polyelectrolyte as the adsorbent material was prepared by using a fast and simple oil–water (ethyl acetate: water) emulsion coagulation method to study the adsorption behavior of cationic methylene blue dye molecules in batch and continuous adsorption setups. The morphological analysis of the Chi-PSS beads shows that the incorporation of PSS into chitosan produces a rougher pore structure due to the binding of PSS polymer chains onto the chitosan backbone structure. In the batch adsorption studies, the chitosan beads incorporated with PSS achieved a maximum adsorption capacity of 42.21 mg/g based on the Langmuir adsorption model. The Langmuir adsorption isotherm and intraparticle diffusion adsorption kinetics provided a favorable fit for the system’s adsorption equilibrium, and thermodynamic parameters demonstrated that the physisorption of methylene blue molecules onto Chi-PSS beads was spontaneous and exothermic in nature, which does not favor adsorption at higher temperatures. In addition, the regeneration of Chi-PSS beads could be performed using pyridine, urea, HCl, oxalic acid, and NaOH, where NaOH demonstrated the highest adsorption capacity after regeneration compared to other reagents due to its higher selectivity over other counter ions. This was predicted, considering that cationic dye molecules have a higher affinity for the cationic part of desorption agents (Na^+^), which allow the methylene blue molecules to be displaced from the PSS surface. Furthermore, the adsorption performance upon regeneration demonstrated good performance with a continuous adsorption setup up until the third cycle, with a total adsorption capacity of 198 mg/g. In this study, we found that chitosan-PSS beads have high potential as a reusable adsorbent material to remove anionic pollutants from aqueous wastewater in batch and continuous water treatment systems.

## Figures and Tables

**Table 1:** Kinetics parameters of the adsorption of methylene blue onto Chi-PSS beads as determined using pseudo-first-order, pseudo-second-order and intraparticle diffusion kinetics model.

Mass of Adsorbent (g)	Pseudo-First-Order			Pseudo-Second- Order			q_e_ exp (mg/g)	Intraparticle Diffusion	
	q_e1_ cal (mg/g)	k_1_	r^2^	q_e2_ cal (mg/g)	k_2_	r^2^		K_p1_	r^2^
0.05	3.95	0.019	0.84	1.78	0.250	0.61	2.99	0.196	0.99
0.1	7.09	0.023	0.88	3.42	0.179	0.77	5.09	0.456	0.99
0.25	11.03	0.062	0.77	5.12	0.229	0.88	6.08	0.857	0.99
0.5	11.18	0.090	0.78	5.12	0.358	0.85	6.16	0.975	0.99

**Table 2:** Parameters of methylene blue adsorption onto Chi-PSS beads as determined using Langmuir, Freundlich, and Temkin adsorption isotherms.

Model	Parameters	
Langmuir isotherm	Q_0_ (mg g^−1^)	42.21
	K_L_ (L mg^−1^)	6.36
	r^2^	0.97
Freundlich isotherm	K_F_	25.73
	n	10.08
	r^2^	0.85
Temkin isotherm	A_T_	2733.35
	b_T_	739.61
	r^2^	0.88

**Table 3:** Thermodynamic parameters for methylene blue adsorption onto Chi-PSSI beads.

Temperature (K)	ΔH (kJ/mol)	ΔS (J/mol)	ΔG (J/mol)
298	−103.66	−295.38	15.57
318			9.87
338			3.74

## Data Availability

No new data were created or analyzed in this study. Data sharing is not applicable to this article.
